# Clinical Obesity and Insulin Resistance Assessed Based on SHBG Levels and HOMA-IR, TyG-Waist Circumference (TyG-WC), TyG-Waist to Height Ratio (TyG-WHtR), and TyG-Waist to Hip Ratio (TyG-WHR) Values in Young Caucasian Women with Polyendocrine Metabolic Ovarian Syndrome (PMOS)

**DOI:** 10.3390/jcm15145696

**Published:** 2026-07-21

**Authors:** Marta Kochanowicz, Aleksander Jerzy Owczarek, Paweł Małek, Paweł Madej, Jerzy Chudek, Magdalena Olszanecka-Glinianowicz

**Affiliations:** 1 Department of Gynecological Endocrinology, Faculty of Medical Sciences in Katowice, Medical University of Silesia in Katowice, 14 Medyków Str., 40-752 Katowice, Poland; marthakoch@interia.pl (M.K.); pmadej@sum.edu.pl (P.M.); 2 Health Promotion and Obesity Management Unit, Department of Pathophysiology, Faculty of Medical Sciences in Katowice, Medical University of Silesia in Katowice, 18 Medyków Str., 40-752 Katowice, Polandpmalek@sum.edu.pl (P.M.); 3 Department of Internal Medicine and Oncological Chemotherapy, Faculty of Medical Sciences in Katowice, Medical University of Silesia in Katowice, 20 Francuska Str., 40-027 Katowice, Poland; chj@poczta.fm

**Keywords:** PMOS, clinical obesity, insulin resistance, TyG-WC, TyG-WHtR, TyG-WHR

## Abstract

**Objectives:** The study aimed to assess (1) the prevalence of clinical obesity in a cohort of young Caucasian women with polyendocrine metabolic ovarian syndrome (PMOS), (2) estimate the cut-off value for triglyceride-glucose index—waist-to-height ratio (TyG-WHtR) and TyG—waist-to-hip ratio (TyG-WHR) indices discriminating the insulin resistance (IR) based on the HOMA-IR values and sex-hormone-binding-globulin (SHBG) levels, as well as (3) the occurrence of IR based on SHBG levels and HOMA-IR, TyG, TyG—body mass index (TyG-BMI), TyG—waist circumference (TyG-WC), TyG-WHtR, and TyG-WHR indices. **Methods:** This analysis included data retrieved from the medical records of 580 consecutive Caucasian women, for the first time, diagnosed with PMOS at the Gynecological Endocrinology Clinic, including anthropometric data, fasting glucose, insulin, and SHBG levels. Obesity and clinical obesity were diagnosed based on the WHO and LDEC criteria, respectively. The cut-off value for TyG-WHtR and TyG-WHR indices was calculated using receiver operating characteristic curve analysis. The occurrence of IR based on seven indices was assessed. **Results:** The prevalence of clinical obesity was 9.8% among women with BMI < 25 kg/m^2^, 70.2% with BMI 25–29.9 kg/m^2^, 100% with BMI > 30 kg/m^2^. The empirical optimal cut-off values for the TyG-WHtR, corresponding to HOMA-IR ≥ 2.1 and SHBG ≤ 43.1 nmol/L, were 4.44 and 4.35, respectively, while for the TyG-WHR, 6.94 and 6.90, respectively. None of the IR indices identified all clinically obese women. SHBG cut-off was the marker that most frequently detected IR in both subgroups with and without clinical obesity. **Conclusions:** 1. The application of a new definition of clinical obesity increases the classification of obesity among women with PMOS. However, further studies are necessary to confirm the cut-off points for indirect anthropometric parameters of excess visceral fat based on DXA. 2. The cut-offs for the TyG-WHtR and TyG-WHR indices discriminating IR in young Caucasian women with PMOS were similar regardless of whether they were based on HOMA-IR and SHBG cut-offs. 3. SHBG concentration can be considered the most valuable marker for the diagnosis of IR in women with PMOS, both with and without clinical obesity.

## 1. Introduction

Polycystic ovary syndrome (PCOS), officially renamed polyendocrine metabolic ovary syndrome (PMOS) in May 2026 [1], is the most common endocrinopathy in women of reproductive age, encompassing a range of endocrine and metabolic disturbances. PMOS is a heterogeneous syndrome both in terms of the underlying disorders and body weight. Genetic predisposition is believed to play a critical role in its development. Currently, PMOS is considered one of the main hormonal complications of obesity, and insulin resistance (IR) caused by excess visceral fat is a key link in hormonal and metabolic disturbances [2]. However, not all women with PMOS are diagnosed as overweight or obese based on BMI [3]. In March 2025, the Commission Lancet Diabetes and Endocrinology (LDEC) formulated a definition and diagnostic criteria for clinical obesity. This Commission defined clinical obesity as a chronic, systemic illness characterized by alterations in the function of tissues, organs, the whole individual, or a combination thereof, resulting from excess adiposity. In accordance with the diagnostic criteria for clinical obesity, the first step is the assessment of excess adiposity based on anthropometric parameters, including waist circumference (WC), waist-to-height ratio (WHtR), or waist-to-hip ratio (WHR), in addition to body mass index (BMI), using validated methods and cut-off points appropriate to age, gender, and ethnicity. In people with BMI < 30 kg/m^2^, additional parameters of excess adiposity are required for the diagnosis of obesity; in people with BMI 30–39.9 kg/m^2^, only one parameter is needed, and in people with BMI > 40 kg/m^2^, excess adiposity measurements are not required for further confirmation. The second step is the assessment of alterations in the function of tissues, organs, and the whole individual resulting from excess adiposity. One of these alterations in women is PMOS [4]. Thus, all women with PMOS meeting the anthropometric criteria will be diagnosed as clinically obese, regardless of BMI. Since studies have shown that excess visceral fat and the associated changes in systemic inflammation and adipokines secretion play a significant role in the pathogenesis of both the development of insulin resistance (IR) and the hormonal and metabolic disorders occurring in PMOS [5,6,7], the concept of anthropometric measurements indirectly reflecting the excess of this adipose tissue included in the LDEC definition of clinical obesity [4] is a better approach to assessing obesity in this group of women than its current diagnosis based on BMI in accordance with the WHO criteria. The prevalence of clinical obesity in the population of women with PMOS has not been analyzed so far.

The main method of assessing IR is the homeostatic model assessment of IR (HOMA-IR). However, in recent years, attention has been drawn to the fact that variability in glucose and insulin concentrations, affected by various factors, may lead to an erroneous assessment of IR [8]. Moreover, methods for measuring insulin may influence HOMA-IR values [9]. Furthermore, no commonly accepted HOMA-IR cut-off points for the diagnosis of IR were established [10,11,12,13]. In a population of young Caucasian women with PMOS, we set the HOMA-IR cut-off point for IR at ≥2.1 [14]. Therefore, new markers of IR are constantly being sought. One of the proposed markers of IR was SHBG level. SHBG is mainly synthesized in the liver, and its synthesis is inhibited by hyperinsulinemia, compensating for IR [15,16,17]. Our previous studies have shown that SHBG levels may serve as a marker of IR in women with PMOS [18]. A recently published diagnostic test accuracy meta-analysis established a cut-off of SHBG ≤ 43.1 nmol/L, with high sensitivity and specificity, enabling the detection of IR [19]. It should be noted that both HOMA-IR and SHBG are primarily markers of hepatic IR. Based on knowledge of metabolic changes occurring in the insulin-resistant liver, including impaired oxidation and utilization of fatty acids and triglycerides, as well as intensified gluconeogenesis and impaired storage of glucose as glycogen [20,21], the triglyceride-glucose (TyG) index was recently proposed [22]. We established that the cut-off point for the TyG index in young Caucasian women with PMOS, which discriminates against IR, is 8.31 [23]. However, some data suggested that better markers of IR than the TyG index alone are indices derived from the TyG index and the anthropometric parameters BMI, WC, WHtR, and WHR [24,25,26]. These indices are calculated by multiplying the TyG index value by the respective anthropometric parameters. Recently, we established that the cut-off points for the TyG-BMI indices based on HOMA-IR and SHBG in young Caucasian women with PMOS that discriminate against IR are 230 and 233, respectively, and for TyG-WC are 735 and 734, respectively [27]. So far, there is a lack of established cut-off points for TyG-WHtR and TyG-WHR in women with PMOS. Therefore, the study aimed to assess (1) the prevalence of clinical obesity in the cohort of young Caucasian women with PMOS, (2) estimate the cut-off value for TyG-WHtR and TyG-WHR indices discriminating IR based on the HOMA-IR values and SHBG levels, as well as (3) the occurrence of IR based on SHBG levels and HOMA-IR, TyG, TyG-BMI, TyG-WC, TyG-WHtR, and TyG-WHR indices.

## 2. Materials and Methods

### 2.1. Study Population

This retrospective study included data from the medical records of 611 consecutive Caucasian women who were first diagnosed with PMOS according to the Rotterdam criteria [28] and hospitalized at the Department of Gynecological Endocrinology in 2019–2023.

The inclusion criteria were the diagnosis of PMOS regardless of phenotype, age 18–40 years, and a complete dataset in medical records necessary for this analysis. The exclusion criteria, based on medical history and laboratory test results, included diagnosis of other endocrinological disturbances, type 1 and 2 diabetes mellitus, hypercholesterolemia, arterial hypertension, and any pharmacological therapy, including oral contraceptives, metformin, antiandrogens, or thyroid medications, non-pharmacological and pharmacological treatment of obesity, and changes in body mass during the last 3 months.

### 2.2. Clinical and Laboratory Data

The dataset included age, body mass, height, waist circumference, and routine measurements of fasting glucose, triglycerides, insulin, DHEA-S, total testosterone, free testosterone, androstenedione, and SHBG; all were measured in a single hospital laboratory using the same methods for all study subjects. Serum glucose and triglyceride concentrations were measured using the colorimetric method (Roche Mannheim, Germany, reagents Cobas c111, test numbers for glucose 4657527190 and triglycerides 04657594190). DHEA-S, total testosterone, free testosterone, androstenedione, SHBG, and insulin levels were determined using the ECLIA method (Roche Diagnostic GmbH, Mannheim, Germany, reagents for Cobas e411, test number for DHEA-S, 03000087122, test number for total testosterone 08946353190, test number for free testosterone 08946353190, test number for androstenedione 1530, test number for SHBG 750, and for insulin 650).

The occurrence of hirsutism was assessed using the Ferriman and Gallwey scale, with a cut-off of >8 points for diagnosis [29]. Acne was assessed based on the global acne severity scale [30].

The cut-off points for hyperandrogenemia were >55 ng/mL for total testosterone and >3.1 ng/mL for androstenedione.

BMI, WHtR, WHR, HOMA-IR, and TyG, TyG-BMI, TyG-WC, TyG-WHtR, and TyG-WHR indices values were calculated with the following standard formulas:
HOMA-IR = fasting insulin level [uIU/mL] × fasting glucose level [mg/dL]/405TyG = Ln {fasting triglycerides level [mg/dL] × fasting glucose level [mg/dL]/2}TyG-BMI = Ln {[fasting triglycerides level [mg/dL] × fasting glucose level mg/dL]/2} × BMITyG-WC = Ln {fasting triglycerides level [mg/dL] × fasting glucose level [mg/dL]/2} × WCTyG-WHtR = Ln {fasting triglycerides level [mg/dL] × fasting glucose level [mg/dL]/2} × WHtRTyG-WHR = Ln {fasting triglycerides level [mg/dL] × fasting glucose level mg/dL/2} × WHR

### 2.3. Data Analysis

During the analysis of medical records of 611 women with PMOS, it was found that 31 of them (5.1%) were diagnosed with thyroid diseases, 2 (0.3%) had type 1 diabetes, 5 (0.8%) had type 2 diabetes mellitus, and 8 (1.3%) had arterial hypertension. In accordance with the exclusion criteria, they were excluded from the analysis. Finally, 580 (94.9%) medical records were analyzed.

The cut-off points for anthropometric parameters to assess excess adiposity, according to LDEC, were set at appropriate thresholds: waist circumference > 80 cm, WHtR > 0.5, and WHR > 0.85. Clinical obesity was diagnosed in women with BMI < 30 kg/m^2^, 2 of 3 additional parameters were equal to or greater than their cut-off points in women with BMI 30.0–39.9 kg/m^2,^ and 1 of 3 additional parameters were equal to or greater than their cut-off points in all women with BMI > 40 kg/m^2^ [4].

The cut-off points for IR were:

- HOMA-IR ≥ 2.1 [14],
- SHBG ≤ 43.1 [19],
- TyG ≥ 8.31 [23],
- TyG-BMI ≥ 230 [27],
- TyG-WC ≥ 735 [27],
- TyG-WHtR established in this study,
- TyG-WHR established in this study.

### 2.4. Statistical Analysis

Statistical analysis was performed using STATISTICA 13.0 PL (TIBCO Software Inc., Santa Clara, CA, USA), StataSE 13.0 (StataCorp LP, College Station, TX, USA), and R software (R version 4.5.1 (2025-06-13 ucrt)—“Great Square Root”; R Core Team 2013; R: A language and environment for statistical computing. R Foundation for Statistical Computing, Vienna, Austria. URL http://www.R-project.org/). Statistical significance was set at a *p*-value < 0.05. All tests were two-tailed. Imputations were not performed for missing data. Nominal and ordinal data were expressed as percentages. Interval data were expressed as median with lower and upper quartiles. The distribution of the variables was evaluated by the W Shapiro–Wilk test and the quantile–quantile (Q-Q) plot. To identify the cut-off point of TyG-WHtR and TyG-WHR indices discriminating the IR based on the HOMA-IR value (≥2.1) and SHBG level (≤43.1 nmol/L), non-parametric receiver operating characteristic (ROC) curves were calculated with the area under the curve (AUC) and corresponding sensitivity (Se), specificity (Sp), positive and negative predictive values (PPV and NPV), as well as with the accuracy of classification. To identify an optimal empirical cut-off point, the Youden Index was maximized, parametrically, assuming normally distributed data in both classes, using bootstrapping (N = 2000 runs) with mean as the aggregation function (package ‘*cutpointr*’ in R; v 1.2.1). A comparison between a binary diagnostic test (for TyG-WHtR and TyG-WHR) based on HOMA-IR and SHBG was performed (using the package ‘*testCompareR*’ in R; v. 1.1.1) with a Bonferroni correction for multiple comparisons.

## 3. Results

### 3.1. Clinical Obesity Occurrence

The occurrence of clinical obesity was found in 28 (9.8%) women with BMI < 25 kg/m^2^, 111 (70.2%) with BMI 25–29.9 kg/m^2^, 107 (100%) with BMI 30–30.9 kg/m^2^, and 28 (100%) with BMI > 40 kg/m^2^. The detailed characteristics of the first step for the diagnosis of clinical obesity are listed in Table 1 (a) and (b).

### 3.2. The Empirical Optimal Cut-Off Values for the TyG-WHtR and TyG-WHR Indices

**Figure 1:**
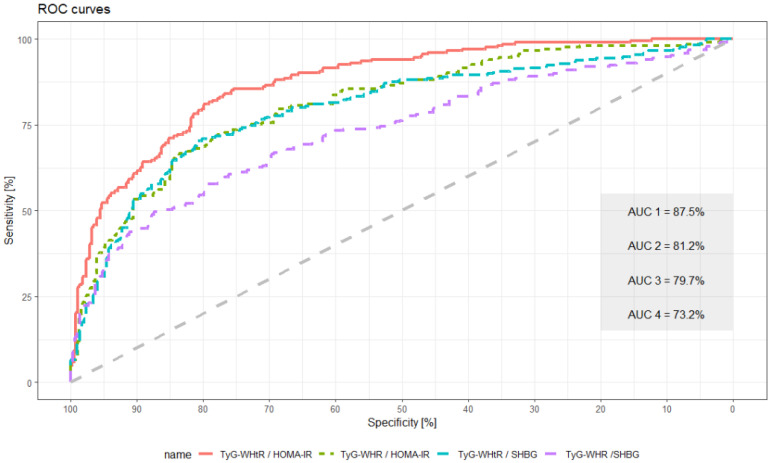
ROC curves for the cut-off points of the TyG-WHtR and TyG-WHR indices for the diagnosis of IR based on HOMA-IR value ≥ 2.1 and SHBG concentration ≤ 43.1 nmol/L; AUC—area under curve.

The empirical optimal cut-off values for the TyG-WHtR index, corresponding to HOMA-IR values ≥ 2.1 and SHBG levels < 43.1 nmol/L, were 4.44 and 4.35, respectively, while for the TyG-WHR index, corresponding to HOMA-IR values ≥ 2.1 and SHBG levels ≤ 43.1 nmol/L, these were 6.94 and 6.90, respectively. The TyG-WHtR index achieves the optimal accuracy, with the highest specificity and both the best positive and negative predictive values. The highest AUC was observed for the TyG-WHtR index using HOMA-IR values ≥ 2.1. The ROC curves are presented in Figure 1.

The clinical credibility based on the calculated cut-off points is presented in Table 2.

**Figure 2:**
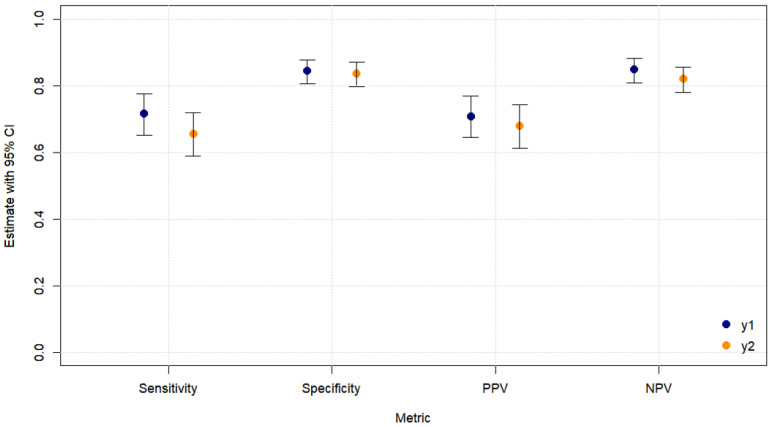
Estimate of clinical diagnostic test measurements for TyG-WHtR (y1) and TyG-WHR (y2) based on HOMA-IR ≥ 2.1.

**Figure 3:**
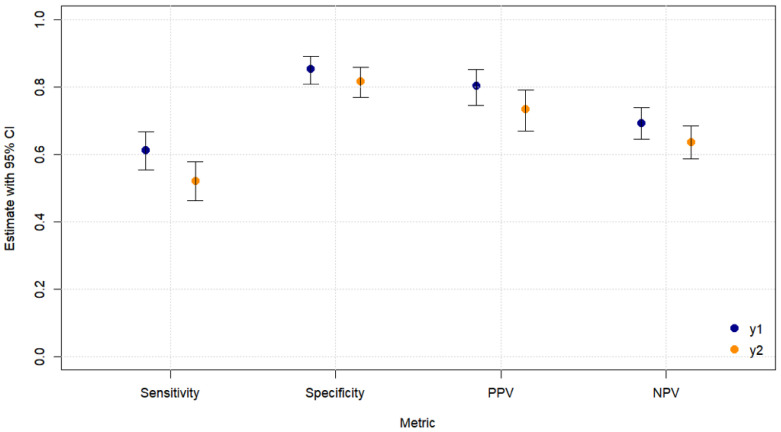
Estimate of clinical diagnostic test measurements for TyG-WHtR (y1) and TyG-WHR (y2) based on SHBG concentration ≤ 43.1 nmol/L.

For both classification variables (HOMA-IR value ≥ 2.1 and SHBG concentration ≤ 43.1 nmol/L), sensitivity (Se) and specificity (Sp), as well as positive/negative predictive values (PPV/NPV; raw and combined, respectively), were compared. There were no differences for HOMA-IR (Se/Sp *p* = 0.347; PPV/NPV *p* = 0.356), while for SHBG levels, for TyG-WHR in comparison to TyG-WHtR, lower values of AUC (*p* < 0.001), sensitivity, and PPV (*p* < 0.05) and NPV (*p* < 0.001) were noted—Figure 2 (for HOMA-IR ≥ 2.1) and Figure 3 (for SHBG concentration ≤ 43.1 nmol/L).

The TyG-WHtR index achieves the optimal accuracy, with the highest specificity and both the best positive and negative predictive values.

**Figure 4:**
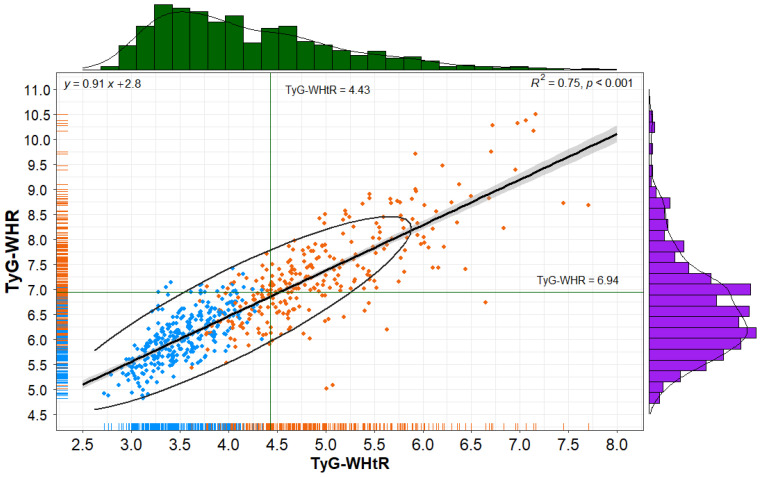
Scatter plot of TyG-WHR versus TyG-WHtR with the regression line. Horizontal and vertical lines indicate the cut-off values derived from the ROC analysis. Orange and blue points represent subjects with and without clinical obesity, respectively. On the sides, histograms of TyG-WHtR (green) and TyG-WHR (purple) are shown with the data distribution line.

There was a very high positive correlation between BMI and TyG-WHtR (r = 0.88; *p* < 0.001) and TyG-WHR (r = 0.60; *p* < 0.001), as well as between the TyG-WHtR and TyG-WHR indices (r = 0.86; *p* < 0.001)—Figure 4.

### 3.3. Clinical and Biochemical Characteristics of PMOS and Occurrence of IR in Women with and Without Clinical Obesity

There were no differences between the percentages of women with phenotypes A, B, C, and D between subgroups with and without clinical obesity (Table 3).

**Figure 5:**
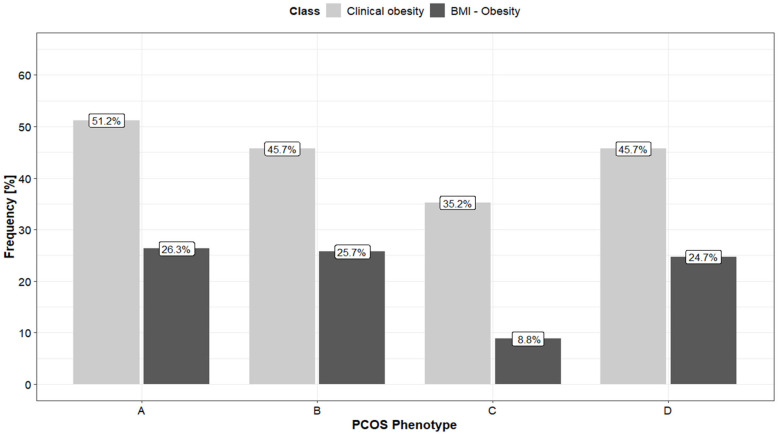
The frequency of clinical obesity and obesity diagnosed according to WHO criteria across PMOS phenotypes.

However, in phenotype A, 51.2% of women had clinical obesity and 26.3% had obesity based on WHO criteria; in phenotype B, 45.7% and 25.7%, respectively; in phenotype C, 35.2% and 8.8%, respectively; and in phenotype D, 45.7% and 24.7%, respectively (Figure 5).

Hirsutism but not acne occurred significantly more often in the subgroup with clinical obesity. Abnormal concentrations of total and free testosterone, as well as DHEA-S, occurred more frequently in the subgroup with clinical obesity. There were no differences in the percentages of women with abnormal androstenedione levels or an elevated LH-to-FSH ratio between subgroups with and without clinical obesity (Table 3).

None of the IR indices used showed IR in all clinically obese women. IR in the subgroup with clinical obesity was most frequently detected when we used SHBG concentrations and TyG-WHtR index as its markers, and least frequently when we used TyG and HOMA-IR. As expected, insulin resistance was found to be significantly less frequent in the subgroup without clinical obesity. In the subgroup without clinical obesity, IR was most frequently detected when SHBG concentrations and the TyG index were used as markers, and least frequently when TyG-WHtR and TyG-WC were applied (Table 3).

In the subgroup with BMI < 25 kg/m^2^ and normal WC, WHtR, and WHR, IR was found in 20.4% based on SHBG levels, in 8.1% based on HOMA-IR, and in 3.4% based on TyG-WHR, while based on the TyG-BMI, TyG-WC, and TyG-WHtR indices, no IR was found in this subgroup. In addition, in this subgroup with one abnormal parameter, including WC, WHtR, and WHR, insulin resistance based on SHBG levels was found in 42.3%, based on TyG-WHR index in 38.5%, based on HOMA-IR in 34.6%, based on TyG index in 23.1%, and based on TyG-WC and TyG-WHtR indices in 11.5%, while based on TyG-BMI index, no IR was found in this subgroup (Table 4 (a)).

In the subgroup with BMI 25–29.9 kg/m^2^ and with normal WC, HHtR and WHR, IR based on SHBG levels was found in 60.9%, based on TyG index in 39.1%, based on HOMA-IR in 13.0% and based on TyG-BMI index in 8.7%, while based on indices TyG-WC, TyG-WHtR, and TyG-WHR, no IR was found in this subgroup. In addition, in this subgroup with one abnormal parameter, including WC, WHtR, and WHR, IR was found in 64.4% based on SHBG levels, 55.6% based on TyG index, 54.8% based on TyG-BMI and TyG-WHtR indices, 52.6% based on TyG-WC index, 51.1% based on TyG-WHR index, and 40.7% based on HOMA-IR (Table 4 (b)).

The frequency of IR in the subgroup with BMI 30–39.9 kg/m^2^, based on anthropometric parameters assessed using individual indices, is presented in Table 4 (c).

## 4. Discussion

To the best of our knowledge, this is the first study that assessed the occurrence of clinical obesity in young Caucasian women with PMOS and estimated cut-off points for TyG-WHtR and TyG-WHR corresponding to IR in women with PMOS.

When Stein and Leventhal first described PCOS in 1935, obesity was considered a symptom of the syndrome [31]. Currently, PMOS is one of the main endocrine complications of obesity [32]. This context has been incorporated into the new definition and diagnostic criteria for clinical obesity. Our study showed that changing the way of diagnosing obesity based on anthropometric parameters other than BMI significantly affects the diagnosis of obesity in a group of young Caucasian women with PMOS. According to WHO criteria [33], obesity was diagnosed in 23.3% of the analyzed group, while, based on LDEC criteria [4], clinical obesity was diagnosed in 47.2% of the analyzed group, including 9.8% with BMI < 25 kg/m^2^, 70.2% with BMI 25–29.9 kg/m^2^, and 100% with BMI > 30 kg/m^2^. Thus, clinical obesity was twice as common as obesity diagnosed based on BMI. However, it should be noted that, when at least one of the three parameters indicated by LDEC was used to diagnose visceral obesity, abnormalities occurred in 8.4% of women with BMI < 25 kg/m^2^ and 15.2% with BMI 25–29.9 kg/m^2^. Therefore, it can be assumed that in another 23.6% of women, the occurrence of excess visceral fat is highly probable. On the other hand, one should consider whether the absence of abnormalities in waist circumference, WHtR, and WHR completely exclude the presence of excess visceral fat depot. Another observation from our study raises doubts because some of the examined women without clinical obesity and with a normal BMI or BMI corresponding to overweight had IR. Failure to diagnose clinical obesity in these women may be due to several factors, including the cut-off points for waist circumference, WHtR, and WHR, as well as measurement errors arising from different individuals performing the measurements. According to LDEC recommendations, the gold standard for assessing excess visceral fat is dual-energy X-ray absorptiometry (DXA) [4]. However, this method is limited in availability and costly to use in everyday clinical practice. Efforts should be made to establish cut-off points for waist and hip circumference based on studies conducted on specific populations using DXA. This would increase the reliability of interpreting anthropometric parameters recommended as indirect indicators of excess visceral fat. Nevertheless, as our study results indicate, the use of the new obesity diagnostic criteria proposed by LDEC [4] has advantages over the previously used WHO criteria [33] in young Caucasian women with PMOS. However, it should be noted that the presence of women with one of the three anthropometric parameters recommended by LDEC among women with BMI < 30 kg/m^2^ raises doubts about whether they should be treated as people without clinical obesity, or whether further research will show that a single parameter would be sufficient. The LDEC framework represents an important conceptual development; its superiority for predicting metabolic outcomes in women with PCOS/PMOS remains to be established, and our study is the first to analyze this concept in this population.

IR resulting from visceral obesity is an important link in the pathogenesis of hormonal disturbances characteristic of PMOS [32]. As described above, in addition to HOMA-IR, other indicators are currently proposed to assess IR [19,22,23,24,25,26,27]. Due to the fact that, according to the diagnostic criteria for clinical obesity, WHtR and WHR are used to assess excess visceral fat beyond waist circumference, and so far, the cut-off points for assessing IR in young Caucasian women with PMOS have been determined only for TyG-WC [27], we also determined cut-off points for TyG-WHtR and TyG-WHR. The empirical optimal cut-off values for the TyG-WHtR index, corresponding to HOMA-IR ≥ 2.1 and SHBG ≤ 43.1 nmol/L, were 4.44 and 4.35, respectively, while for the TyG-WHR index, corresponding to HOMA-IR values ≥ 2.1 and SHBG ≤ 43.1 nmol/L, the values were 6.94 and 6.90, respectively. The TyG-WHtR index achieves the optimal accuracy, with the highest specificity and both the best positive and negative predictive values. The cut-off points estimated in our study were lower than those estimated previously in 1,727 adults with MAFLD from the 2017–2018 National Health and Nutrition Examination Surveys (4.44 and 4.35 vs. 4.94) [26]. The AUC for cut-off points for TyG-WHtR estimated in our study, based on HOMA-IR, was higher, and, based on SHBG, slightly lower than in the NHANES population (87.5% and 79.7% vs. 80.9%) [26]. The cut-off points for TyG-WHtR estimated in our study were also lower than those estimated in 12,309 adults aged 18–60 years, with and without hypertension, from the NHANES 2009–2018 cohort (4.44 and 4.35 vs. 4.83), while the AUCs were higher (87.5% and 79.7% vs. 69.5%) [34]. So far, there is a lack of studies that assessed the cut-off point for TyG-WHtR to assess IR in women with PMOS. Despite numerous studies evaluating the TyG-WHR index as a marker of MAFLD, cardiovascular risk factors, and mortality risk factors, there is no data on its cut-off points in the populations analyzed. In our study, we determined two cut-off points for TyG-WHtR and TyG-WHR. Both cut-off points, determined using HOMA-IR, were more sensitive, but the specificity of the TyG-WHtR cut-off based on SHBG concentration was higher than that based on HOMA-IR. In addition, PPV was higher, and NPV was lower for both cut-off points estimated for SHBG levels. It should be noted that cut-offs for TyG-WHtR and TyG-WHR were not validated against the gold standard for IR assessment (hyperinsulinemic-euglycemic clamp).

The next aim of our analysis was to examine the clinical and biochemical characteristics of the women studied according to the presence or absence of clinical obesity. Of interest, we did not observe differences in the percentage of clinical obesity across phenotypes (A–D). Some previously published studies have suggested that obesity diagnosed based on BMI occurred more frequently among women with phenotypes A and B [35,36]. However, analyzing the frequency of clinical obesity and obesity diagnosed according to WHO criteria shows that both types occur least frequently in phenotype C. Moreover, regardless of PMOS phenotype, the change in how obesity is diagnosed resulted in a nearly twofold increase in its diagnosis among women with phenotypes A, B, and D, and a nearly fourfold increase among women with phenotype C. It should also be noted that both hirsutism and hyperandrogenemia occur frequently in a subgroup with clinical obesity. Since the main link between obesity and PMOS is IR, we assessed the prevalence of IR using seven markers. One would expect that IR would be present in all women with clinical obesity and absent in the subgroup without it. However, none of the IR indices used showed IR in all clinically obese women. This may be because none of the currently proposed indirect markers of IR are sufficiently sensitive for early diagnosis when used on a single occasion. It appears that all proposed indices, except for SHBG concentration, require careful standardization of their component measurements and multiple assessments. In turn, each of these indicators showed the presence of IR in the subgroup without clinical obesity. As mentioned above, this may be due to excess visceral fat in women with a BMI < 30 kg/m^2^ who did not meet the LDEC criteria for clinical obesity. This indicates the need for a study in which excess fat will be assessed with DXA. Imaging studies are also necessary to assess not only visceral fat area but also liver steatosis, and to evaluate their impact on the values of the proposed indirect markers of IR and their cut-off points. However, in a part of lean women with PMOS, insulin resistance may be associated with probably genetically determined post-receptor insulin resistance [37]. Pre-receptor mechanisms also cannot be ruled out. Both of these aspects require further studies.

The lack of diagnosis of IR in all women with clinical obesity may indicate the need to lower the cut-off points of all analyzed markers. Although it should be emphasized that their cut-off points for the diagnosis of IR adopted in our study were lower than those indicated by other researchers for general populations: HOMA-IR < 2.1 and for the general population 2.5 [10], SHBG 43.1 nmol/L and for the general population 50 nmol/L [38], TyG-BMI 230 and for general population 238 [26], TyG-WC 735 and for general population 822 [26], and TyG-WHtR 4.43 and for general population 4.94 [26]. Only the cut-off point for TyG of 8.31 was higher than estimated for the general population of 4.65 [22] but was lower than estimated for Asian women with PMOS as 8.51 [39]. No cut-off point has been established for the general population for TyG-WHR. On the other hand, other explanations for these results should also be considered, including biological heterogeneity, limitation of anthropometric classification, and duration of obesity, which may be significant in the development of IR, as well as factors such as regular physical activity, which may delay the development of IR in some people with obesity.

In both subgroups with and without clinical obesity, the SHBG level seems to be the most valuable marker for the diagnosis of IR. Of interest, in the subgroup with clinical obesity, IR occurred least frequently when we used TyG and HOMA-IR to evaluate it, while in the group without clinical obesity, the TyG index was the second marker after SHBG in terms of the frequency of detecting IR. This may be due to the greatest stability of the determined SHBG levels compared to the concentrations of insulin, glucose, and triglyceride levels needed to calculate other markers of IR, as was shown by Jaygobal et al. [8] in 10 samples obtained every 4 days from the same persons. However, it should be noted that circulating androgen excess and estrogen levels are the factors that may limit its specificity. Therefore, it would require confirmation by the hyperinsulinemic-euglycemic clamp.

Among the two new markers whose cut-off points we determined in our study, the TyG-WHtR index allowed for the detection of IR with the second highest frequency after SHBG in the subgroup with clinical obesity, while in the subgroup without clinical obesity, next to TyG-WC, it was one of the indicators that diagnosed it the least frequently. This may be due to the variability of glucose and triglyceride levels but also indicates the need to assess IR using more than one indicator. It should also be emphasized that the separate use of indirect markers of IR requires further verification and detailed studies before guidelines for their use in everyday clinical practice can be established. It should also be noted that all analyzed markers are associated with hepatic IR.

Our study has several limitations. The main limitation is its retrospective design. It also lacks assessment of body composition by DXA and of fatty liver by FibroScan or magnetic resonance. Furthermore, due to the retrospective nature of the study, small errors in anthropometric measurements cannot be ruled out, as measurements were not always performed by the same person, and the subjects were not specifically instructed to stand upright with the abdominal muscles relaxed and to exhale calmly during the measurement. It should be noted, however, that the clinic staff performing waist and hip circumference measurements were trained in the appropriate locations in accordance with WHO criteria [40].

The strength of our study relies on the large size of the study group and the inclusion of a homogenous cohort of young Caucasian women with PMOS and a wide range of BMI. Of note, our study is the first to apply the definition of clinical obesity in women with PMOS. Furthermore, we demonstrated that using a single IR marker may lead to a missed diagnosis. Our results suggest the need for large, multicenter studies to determine how to diagnose IR in women with PMOS in daily clinical practice.

## 5. Conclusions

1. The application of a new definition of clinical obesity increases the classification of obesity among women with PMOS. However, further studies are necessary to confirm the cut-off points for indirect anthropometric parameters of excess visceral fat based on DXA.

2. The cut-offs for the TyG-WHtR and TyG-WHR indices discriminating IR in young Caucasian women with PMOS were similar regardless of whether they were based on HOMA-IR values or SHBG levels.

3. SHBG concentration can be considered the most valuable marker for the diagnosis of IR in women with PMOS, both with and without clinical obesity.

## Figures and Tables

**Table 1:** (**a**) The detailed characteristics of the first step in diagnosing clinical obesity among women with normal BMI and BMI corresponding to overweight according to WHO criteria. (**b**) The detailed characteristics of the first step in diagnosing clinical obesity among women with BMI corresponding to classes I and II according to WHO criteria.

(**a**)									
	**WC + WHtR**	**WC + WHR**	**WHtR + WHR**	**One of Three**		**All Three**		**None of the Three**	
BMI < 25 kg/m^2^ N (%) 287 (49.5)	12 (4.2)	3 (1.0)	0	24 (8.4)		13 (4.5)		235 (81.9)	
BMI 25–29.9 kg/m^2^ N (%) 158 (27.2)	61 (38.6)	1 (0.6)	0	24 (15.2)		49 (31.0)		23 (14.6)	
(**b**)									
	**WC**	**WHtR**		**WHR**			**None of the three**		
BMI 30–39.9 kg/m^2^ N (%) 107 (18.4)	107 (100)	106 (99.1)		60 (56.1)			0		

**Table 2:** Results of the ROC curve analysis and clinical credibility based on the calculated cut-off points.

	Based on HOMA-IR ≥ 2.1		Based on SHBG Levels ≤ 43.1 nmol/L	
	TyG-WHtR	TyG-WHR	TyG-WHtR	TyG-WHR
Area under curve (%)	87.5 (84.5–90.4)	81.2 ^#^ (77.5–84.9)	79.7 (76.1–83.4%)	73.2 ^#^ (69.1–77.3)
Cut-off value	4.4297	6.9369	4.3497	6.9039
Accuracy (%)	80.0	77.41	73.45	67.07
Sensitivity (%)	71.6 (64.8–77.6)	65.7 (58.6–72.1)	61.2 (55.2–66.8)	52.1 ^#^ (46.1–58.0)
Specificity (%)	84.4 (80.3–87.8)	83.6 (79.4–87.1)	85.3 (80.7–89.1)	81.6 (76.6–85.8)
Positive predictive value (%)	70.9 (64.1–77.0)	68.0 (60.9–74.4)	74.2 (68.1–85.2)	73.4 * (66.7–79.2)
Negative predictive value (%)	84.9 (80.8–88.3)	82.1 (77.8–85.7)	69.3 (64.3–74.0)	63.7 ^#^ (58.6–68.5)
Diagnostic odds ratio	13.70 (9.06–20.72)	13.04 (8.65–19.65)	9.20 (6.16–13.75)	4.83 (3.32–7.04)
F1 score	0.713	0.668	0.694	0.609
*p*-value	<0.001	<0.001	<0.001	<0.001
True positive	144	132	175	149
False negative	57	69	111	137
False positive	59	62	43	54
True negative	320	317	251	240

TyG-WHR vs. TyG-WHtR: * *p* < 0.05, ^#^ *p* < 0.001.

**Table 3:** Clinical and biochemical characteristics of PMOS and occurrence of insulin resistance.

	With Clinical Obesity N (%) 274 (47.2)	Without Clinical Obesity N (%) 306 (52.8)	*p*-Value
PMOS phenotypes [N; (%)]			
A	173 (63.1)	165 (53.9)	0.06
B	32 (11.7)	38 (12.4)	
C	32 (11.7)	59 (19.3)	
D	37 (13.5)	44 (14.4)	
Hirsutism [N; (%)]	120 (43.8)	79 (25.8)	<0.001
Acne [N; (%)]			
No	110 (40.1)	107 (35.0)	0.18
Yes	164 (50.9)	199 (65.0)	
Hyperandrogenemia [N; (%)]			
Total testosterone > 0.48 nmol/L [N; (%)]	95 (34.7)	48 (15.7)	<0.001
Free testosterone > 2.85 pg/mL [N; (%)]	156 (56.9)	81 (26.5)	<0.001
Androstenedione > 3.3 ng/mL [N; (%)]	33 (12.0)	33 (10.8)	0.63
DHEA-S > 370 ug/dL [N; (%)]	98 (35.8)	81 (26.5)	<0.05
LH/FSH > 1.5 [N; (%)]	72 (26.3)	90 (29.4)	0.04
Insulin resistance based on [N; (%)]			
• HOMA-IR > 2.1	166 (60.6)	35 (11.4)	<0.001
• SHBG < 43.1 nmol/L	204 (74.4)	82 (26.8)	<0.001
• TyG > 8.31	163 (59.5)	76 (24.8)	<0.001
• TyG-BMI > 230	199 (72.6)	12 (3.9)	<0.001
• TyG- WC > 735	195 (71.2)	4 (1.3)	<0.001
• TyG-WHtR > 4.43	202 (73.7)	1 (0.3)	<0.001
• TyG-WHR > 6.94	176 (64.2)	18 (5.9)	<0.001

**Table 4:** (**a**) The frequency of insulin resistance according to the anthropometric parameters in the subgroup with BMI < 25 kg/m^2^, N = 287 (49.5%). (**b**) The frequency of insulin resistance according to the anthropometric parameters in the subgroup with BMI 25–29.9 kg/m^2^, N = 158 (27.2%). (**c**) The frequency of insulin resistance according to the anthropometric parameters in the subgroup with BMI 30–39.9 kg/m^2^, N = 107 (18.4%).

(**a**)							
**Insulin** **Resistance**	**HOMA-IR ≥ 2.1** **[N (%)]**	**SHBG ≤ 43.1 nmol/L** **[N (%)]**	**TyG ≥ 8.31** **[N (%)]**	**TyG-BMI ≥ 230** **[N (%)]**	**TyG-WC ≥ 735** **[N (%)]**	**TyG-WHtR ≥ 4.43** **[N (%)]**	**TyG-WHR ≥ 6.94** **[N (%)]**
**Prevalence of visceral obesity parameters [N (%)]**							
**WC + WHtR** **N = 12 (4.2%)**	5 (41.7)	6 (50.0)	1 (8.3)	0	0	0	1 (8.3)
**WC + WHR** **N = 3 (1.0%)**	0	0	1 (33.3)	0	0	0	2 (66.7)
**WHtR + WHR** **N = 0**	0	0	0	0	0	0	0
**One of three** **N = 24 (8.4%)**	18 (34.6)	22 (42.3)	12 (23.1)	0	6 (11.5)	6 (11.5)	20 (38.5)
**All three** **N = 13 (4.5%)**	6 (46.1)	5 (38.5)	3 (23.1)	0	5 (38.5)	6 (46.2)	10 (76.9)
**None of the three** **N = 235 (81.9%)**	19 (8.1)	48 (20.4)	48 (20.4)	0	0	0	8 (3.4)
(**b**)							
**Insulin** **resistance**	**HOMA-IR ≥ 2.1** **[N (%)]**	**SHBG ≤ 43.1 nmol/L** **[N (%)]**	**TyG ≥ 8.31** **[N (%)]**	**TyG-BMI ≥ 230** **[N (%)]**	**TyG-WC ≥ 735** **[N (%)]**	**TyG-WHtR ≥ 4.43** **[N (%)]**	**TyG-WHR ≥ 6.94** **[N (%)]**
**Prevalence of visceral obesity parameters [N (%)]**							
**WC + WHtR** **N = 61 (38.6%)**	28 (45.8)	42 (68.9)	39 (63.9)	38 (62.3)	28 (45.9)	34 (55.7)	23 (37.7)
**WC + WHR** **N = 1 (0.6%)**	0	0	0	0	0	0	0
**WHtR + WHR** **N = 0**	0	0	0	0	0	0	0
**One of three** **N = 24 (15.2%)**	55 (40.7)	36 (73.5)	75 (55.6)	74 (54.8)	71 (52.6)	74 (54.8)	42 (85.7)
**All three** **N = 49 (31.0%)**	21 (42.9)	36 (73.5)	24 (49.0)	26 (53.1)	40 (81.6)	39 (79.6)	42 (85.7)
**None of the three** **N = 23 (14.6%)**	3 (13.0)	14 (60.9)	9 (39.1)	2 (8.7)	0	0	0
(**c**)							
**Insulin** **resistance**	**HOMA-IR ≥ 2.1** **[N (%)]**	**SHBG ≤ 43.1 nmol/L** **[N (%)]**	**TyG ≥ 8.31** **[N (%)]**	**TyG-BMI ≥ 230** **[N (%)]**	**TyG-WC ≥ 735** **[N (%)]**	**TyG-WHtR ≥ 4.43** **[N (%)]**	**TyG-WHR ≥ 6.94** **[N (%)]**
**Prevalence of visceral obesity parameters [N (%)]**							
**WC** **N = 107 (100%)**	80 (74.8)	89 (83.2)	74 (69.2)	107 (100)	94 (87.8)	98 (88.8)	75 (70.1)
**WHtR** **N = 106 (99.1%)**	79 (74.5)	88 (83.0)	73 (68.9)	106 (100)	94 (88.7)	95 (89.6)	75 (70.7)
**WHR** **N = 60 (56.1%)**	50 (83.3)	52 (86.7)	49 (81.7)	60 (100)	60 (100)	58 (96.7)	59 (98.3)

## Data Availability

The data are available from the corresponding author upon request.

## Abbreviations

The following abbreviations are used in this manuscript:

AUC	Area Under the Curve
BMI	Body Mass Index
DHEA-S	Dehydroepiandrosterone Sulfate
DXA	Dual-Energy X-ray Absorptiometry
F1 score	A Measure of Predictive Performance
FN4	False Negatives
FP1	False Positives
FSH	Follicle-Stimulating Hormone
HOMA-IR	The Homeostatic Model Assessment of Insulin Resistance
IR	Insulin Resistance
LDEC	The Lancet Diabetes and Endocrinology Commission
LH	Luteinizing Hormone
MAFLD	Metabolic-Associated Fatty Liver Disease
NPV	Negative Predictive Value
PCOS	Polycystic Ovary Syndrome
PMOS	Polyendocrine Metabolic Ovarian Syndrome
PPV	Positive Predictive Value
ROC	Receiver Operating Characteristic
Se	Sensitivity
SHBG	Sex Hormone-Binding Globulin
Sp	Specificity
TN3	True Negative
TP2	Total Positivity of Order
TyG	Triglyceride Glucose Index
TyG-BMI	Triglyceride Glucose—Body Mass Index
TyG-WC	Triglyceride Glucose—Waist Circumference Index
TyG-WHtR	Triglyceride Glucose—Waist to Height Ratio Index
TyG-WHR	Triglyceride Glucose—Waist to Hip Ratio Index
WC	Waist Circumference
WHO	World Health Organization
WHR	Waist to Hip Ratio
WHtR	Waist to Height Ratio
